# Offset-decoupled deformable convolution for efficient crowd counting

**DOI:** 10.1038/s41598-022-16415-9

**Published:** 2022-07-18

**Authors:** Xin Zhong, Jing Qin, Mingyue Guo, Wangmeng Zuo, Weigang Lu

**Affiliations:** 1 Department of Educational Technology, Ocean University of China, Qingdao, 266100 China; 2 Department of Computer Science and Technology, Harbin Institute of Technology, Harbin, 150001 China; 3 Department of Electrical and Communication Engineering, University of Chinese Academy of Sciences, Beijing, 100049 China

**Keywords:** Computer science, Information technology

## Abstract

Crowd counting is considered a challenging issue in computer vision. One of the most critical challenges in crowd counting is considering the impact of scale variations. Compared with other methods, better performance is achieved with CNN-based methods. However, given the limit of fixed geometric structures, the head-scale features are not completely obtained. Deformable convolution with additional offsets is widely used in the fields of image classification and pattern recognition, as it can successfully exploit the potential of spatial information. However, owing to the randomly generated parameters of offsets in network initialization, the sampling points of the deformable convolution are disorderly stacked, weakening the effectiveness of feature extraction. To handle the invalid learning of offsets and the inefficient utilization of deformable convolution, an offset-decoupled deformable convolution (ODConv) is proposed in this paper. It can completely obtain information within the effective region of sampling points, leading to better performance. In extensive experiments, average MAE of 62.3, 8.3, 91.9, and 159.3 are achieved using our method on the ShanghaiTech A, ShanghaiTech B, UCF-QNRF, and UCF_CC_50 datasets, respectively, outperforming the state-of-the-art methods and validating the effectiveness of the proposed ODConv.

## Introduction

Public safety has attracted significant attention in recent years due to the increased worldwide population and accelerated urbanization. According to the needs of public safety and transport management, crowd counting^1–3^ in public areas such as campuses, shopping malls, and train stations within a certain range is an essential and challenging task. Furthermore, benefiting from the advancement of digital monitoring^4–6^, the foundation of hardware is provided for research based on crowd counting. Therefore, crowd counting has attracted great attention in the field of computer vision due to the obvious requirement for public security and a stable hardware foundation.

The present crowd counting algorithms can be divided into three types: tracking-based methods^7,8^, feature-based regression methods^9,10^, and CNN-based methods^11–13^. Although superiority in terms of accuracy, efficiency, and robustness is shown in CNN-based approaches, CNNs’ inability to adapt to head-scale changes is still an obstacle, limiting the increase in accuracy. Many CNN-based methods^14,15^ attempt to address the aforementioned problem. However, there are also some barriers to handle. One is that, along with the improved accuracy, the number of arguments in the network has significantly increased, which means the training efficiency is consequently reduced. On the other hand, these methods are deficient in mechanisms to obtain the head-scale features completely by handling geometric transformations.

**Figure 1:**
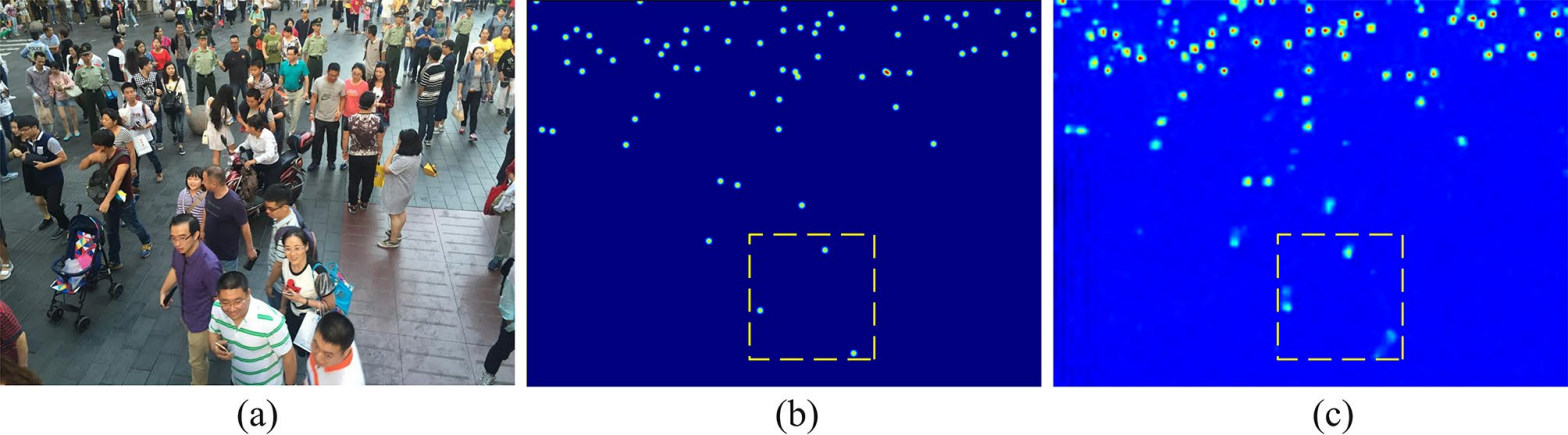
Visualization of density maps predicted from models trained with DConv. (**a**) is one of the input images in the ShanghaiTech B dataset, (**b**) is the ground truth, and the estimated density map is shown in (**c**) which shows less regular Gaussian blobs.

To address the abovementioned problems, a module called deformable convolution (i.e., DConv)^16,17^ is proposed to improve the CNNs’ capability of modeling geometric transformations by adding additional offsets, enhancing the adjustable receptive field of convolution. However, as shown in Fig. 1, when the network is randomly initialized, the parameters of offset convolution are also randomly generated so that the offsets generated by the deformable convolution fluctuate strongly. The sampling points of the deformable convolution are disorderly stacked, which weakens the feature sampling ability. Ultimately, the potential of deformable convolution is not being fully exploited. Based on the preceding discussion, it is difficult to improve the feature extraction ability by simply integrating deformable convolution into the network. Therefore, in this work, the appropriate methods of offset learning are explored.

In this paper, a novel deformable convolution called offset-decoupled deformable convolution (i.e., ODConv) is proposed. To implement this ODConv, the traditional offset map is decoupled into the product of an initial offset map (pre_offset map) and a scale map. The potential performance of deformable convolution is better exploited by controlling the pre_offset map and the scale map. In our implementation, CSRNet^18^ is adopted as the backbone. The last dilated convolution layer is replaced with deformable convolution as the baseline, which is denoted as CSRNet⊕ . Without bells and whistles, by replacing the DConv in CSRNet⊕ with the proposed ODConv, we achieve better performance (2.8%, 7.8%, 3.8%, and 8.8% improvement on ShanghaiTech A, ShanghaiTech B, UCF-QRNF, and UCF_CC_50).

In summary, the main contributions of this paper are twofold. First, an offset-decoupled deformable convolution (ODConv) is proposed, which constrains the offsets to a certain extent. It can obtain more complete information within the effective region of the sampling point while performing the same computation as CSRNet^18^. On the other hand, based on the proposed ODConv, better performance is gained over the state-of-the-art on several crowd counting networks.

## Related work

Since a method is proposed to improve the crowd counting performance by integrating offset-decoupled deformable convolution into the end-to-end network, the following two aspects related to our work are discussed.

### Crowd counting

Crowd counting methods can be classified into three categories: detection-based methods, feature-based regression methods, and CNN-based methods. A sliding window detector is utilized to identify targets in detection-based methods^7,8^ and works well for low-density crowds. By learning a mapping between captured features and counts, regression-based methods^9,10^ have significant advantages over detection-based methods in dealing with occlusion in dense crowds. Furthermore, with the great success of CNNs in other fields, they are widely utilized in crowd counting. In CNN-based methods, input images are mapped to density maps by using a nonlinear function to obtain estimated counts^19–21^. Wang et al*.*^22^ were pioneers in using CNNs to estimate crowd counts, proposing an end-to-end CNN model for counting from images of dense crowds. In contrast to the abovementioned model, Zhang et al*.*^23^ extended the network to a multicolumn architecture for variation of the receptive field and better robustness. On the basis of a multicolumn architecture, Onoro et al*.*^24^ proposed a model called HydraCNN, which is able to adapt to varied crowd counts and scenarios. The similarity between the abovementioned methods is that they all aim to increase the accuracy of crowd counting by altering the structure of the network. Another approach that is used to obtain better performance is to remove the limitation caused by the fixed geometric structures of convolution. For example, CSRNet^18^ was proposed to expand the receptive field while maintaining resolution, and a dilated convolution neural network was used as the back-end network. Nevertheless, CSRNet still has a fixed receptive field for extracting different head-scale sizes of features, resulting in low counting accuracy on various scales.

Considering that spatial information is ignored in the abovementioned methods, there are several approaches to address this issue. One approach is to strengthen the architecture of convolutional neural networks. Yan et al*.*^25^ proposed a perspective-guided convolution network (PGCNet) with an effective perspective estimation branch that guides variation smoothing of feature maps, enhancing the capability of feature alignment. Xia et al*.*^26^ proposed a coordinated feature fusion network (CFFNet) to solve the problem that spatial misalignment is ignored in feature extraction in traditional networks. A module called the spatial alignment module (SAM) was embedded to learn the offset of pixels to generate a high-quality density map for estimation and more detailed spatial distribution descriptions.

### Deformable convolution

In addition to integrating modules and branches in the network to handle scale variations, there is another way to increase the flexibility of feature aggregation, that is, improving the fixed geometric structures of CNNs using deformable convolution^16,17^, which can be used to model nonrigid objects by adding extra learnable offsets to the original model. When 2D offsets, which are dependent on the input features, are added to the regular grid sampling locations in the standard convolution, the sampling grid is enabled to deform freely. Due to the effective improvement in CNNs’ capability of modeling geometric transformations, deformable convolution is widely used in image classification and pattern recognition. For example, Wu et al*.*^27^ proposed an offset-adjustable deformable convolution for virtual tracking. Offset adaptive technology is integrated to capture the spatial information of tracking objects and better performance is achieved. Guo et al*.*^28^ proposed a model called the dilated-attention-deformable convolution network (DADNet), in which deformable convolutional density map estimation (DME) enhances the flexibility of the sampling location of objects by adaptive offsets. Although deformable convolution can be used to model the features of a continuous scale, the offsets of sampling points are difficult to effectively learn, which leads to clustering in the features of sample points. As a result, the potential performance of deformable convolution is unable to be exerted.

As mentioned above, multiscale feature fusion is utilized in most algorithms, which leads to a significant increase in network parameters and computation. However, offsets are relatively large and generally exceed 1 pixel. If only the tanh function is added as an activation function, the offset loss is changed from -1 to 1, indicating that not all offsets are trained effectively. The current methods do not discuss which kind of activation function should be used after offset loss. Therefore, a step-by-step decreasing scaled deformable loss is proposed in this paper to decouple the offset of the deformable convolution into the product of the initial offset and the scale, which constrains the learning of the initial offset and the scale through additional loss. As a result, the deformable convolution’s potential is better utilized.

## Proposed method

In this section, we first introduce the principles of offset-decoupled deformable convolution (i.e., ODConv), and then the architecture of the training network is presented.

### Offset-decoupled deformable convolution

**Figure 2:**
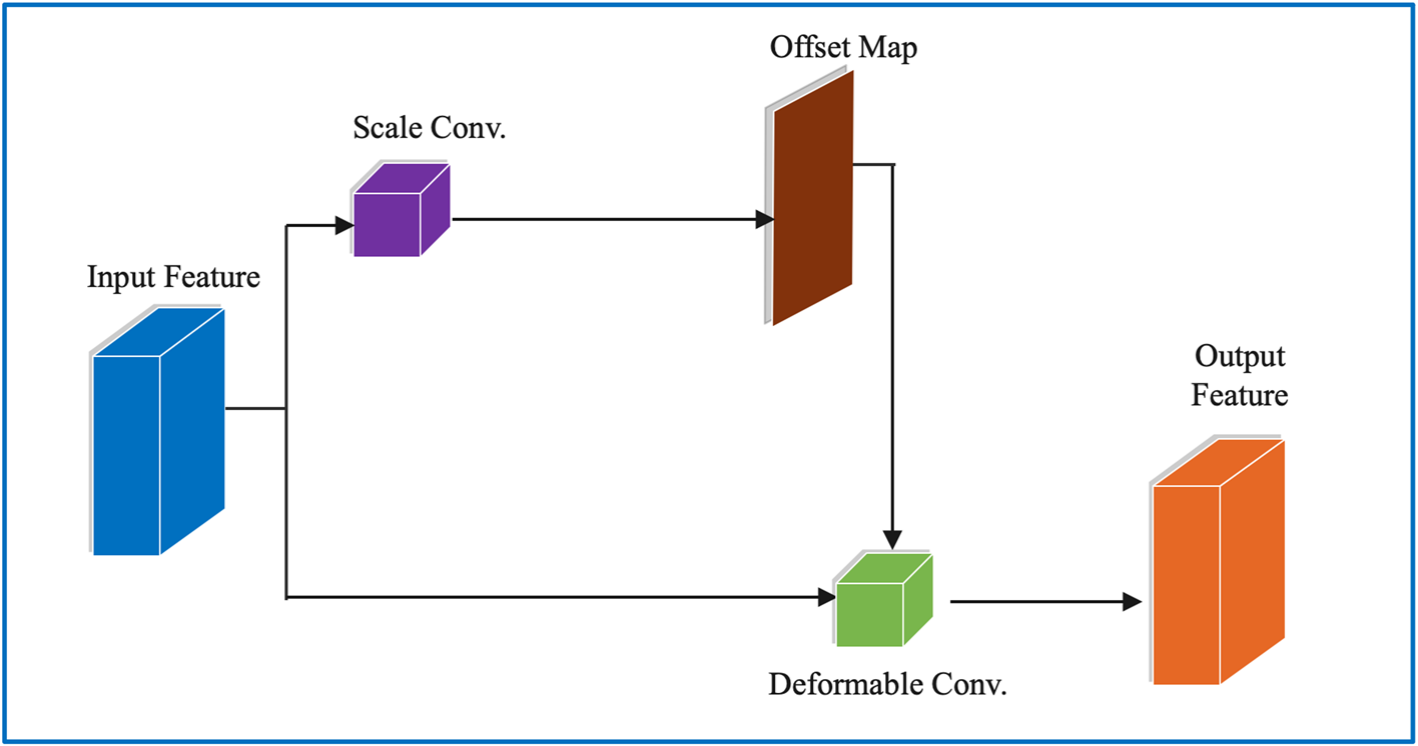
An illustration of a conventional DConv, in which the offsets are obtained directly from the input feature.

As illustrated in Fig. 2, through the computation of offset convolution, the offsets of the traditional deformable convolution are obtained directly from the input feature. Unlike with the conventional deformable convolution, the offsets of the offset-decoupled deformable convolution are obtained from the product of the pre_offset map and the scale map.

**Figure 3:**
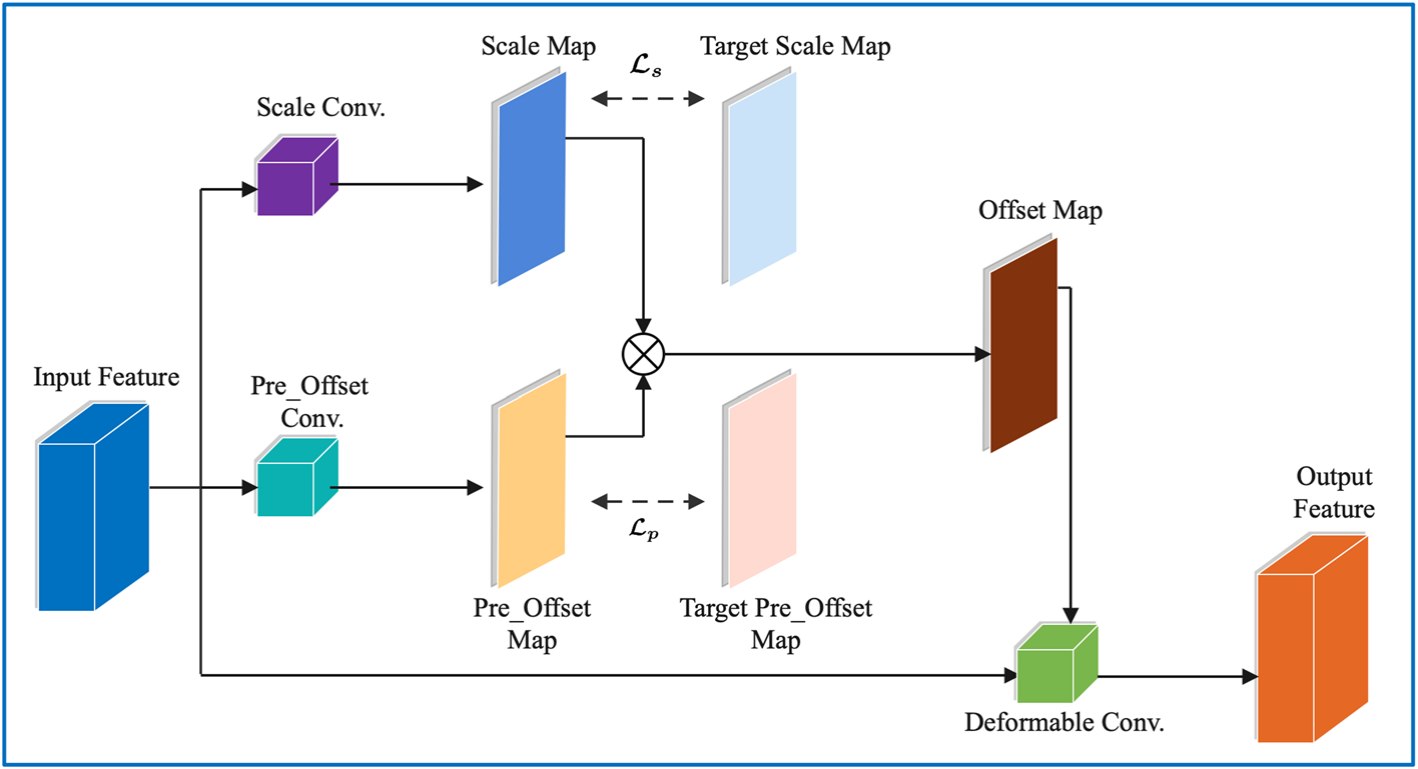
Illustration of our ODConv. The scale map and the pre_offset map are represented by blue and orange parallelograms, respectively. The offsets are obtained from the product of the pre_offset map and the scale map.

As shown in Fig. 3, the pre_offset map and the scale map, which are derived from pre_offset convolution and scale convolution, respectively, are multiplied to obtain the offset map.

For example, given an input feature of size $c\times h\times w$, $c$ is the number of channels, and the scale map, target scale map, pre_offset map, and target pre_offset map are denoted as $S$, ${S}_{t}$, $Off$, and ${Off}_{t}$, respectively. The sizes of the scale map, the pre_offset map, and the offset map are all $18\times h\times w$ (that is, there are offsets of 9 sampling points corresponding to each spatial location).

Thus, the constrained loss of the scale map can be presented as scale loss:

1 $${\mathcal{L}}_{s}=\frac{1}{2N}{\parallel S-{S}_{t}\parallel }_{2}^{2}$$

Then, the loss of the pre_offset map called the pre_offset loss is:

2 $${\mathcal{L}}_{p}=\frac{1}{2N}{\parallel Off-Of{f}_{t}\parallel }_{2}^{2}$$

where each value of ${S}_{t}$ is initialized to 1 and the value of $Of{f}_{t}$ is set to 0. In addition, to ensure that the value of the pre_offset map is between − 1 and 1, the tanh activation function is used after pre_offset convolution. There is also an activation function called ReLU after scale convolution to make the value of the scale map nonnegative. As a result, the value of the offset map has a larger value space.

The loss of density estimation is:

3 $${\mathcal{L}}_{den}=\frac{1}{2N}|P\left({I}_{i};\Theta \right)-{Y}_{i}{|}_{2}^{2}$$

${Y}_{i}$ is the density map of the ground truth, ${I}_{i}$ is the input picture, and $N$ is the batch size. $\Theta$ devotes the parameter of the network, and the estimation of the density map is represented as $P\left({I}_{i};\Theta \right)$. Finally, the total loss is shown as:

4 $${\mathcal{L}}_{\text{total}}=\lambda \left({\mathcal{L}}_{s}+{\mathcal{L}}_{p}\right)+{\mathcal{L}}_{den}$$

In conventional deformable convolution, the learned effect of offsets is completely determined by the input feature and random initialized offset convolution. In this situation, the variance of offsets is so large at some positions on the offset map that the sampling points are disordered. As a result, it is difficult for the network to train.

**Figure 4:**
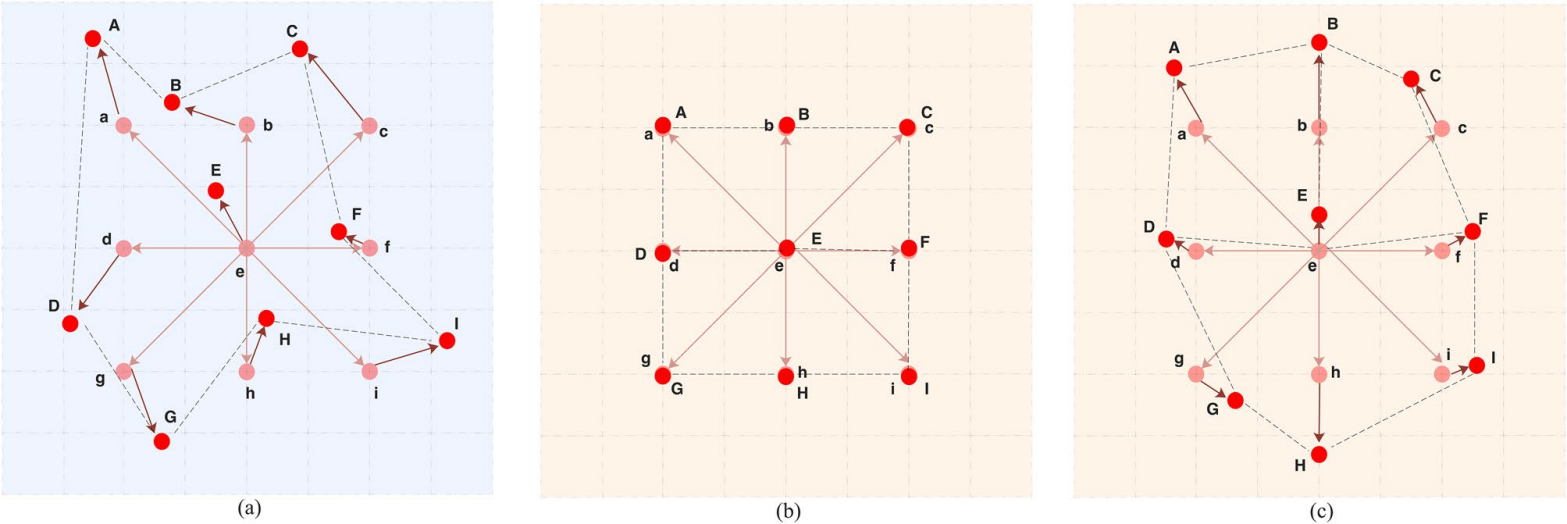
Conventional offset-based deformable convolution is presented in (**a**), and illustrations of the learning process of offsets in offset-decoupled deformable convolution are shown in (**b**) and (**c**). The sampling points are represented by the balls. Among them, the colors of typical convolution sampling points (a–i) and the actual sampling points (A–I) are pink and red, respectively. In addition, offsets are indicated by the dark red arrows.

The abovementioned problem can be addressed by offset-decoupled deformable convolution. Figure 4 shows the comparison between offset-decoupled deformable convolution and conventional offset-based deformable convolution. The sampling points that move with the sampling offsets in Deformable Convolution of kernel size 3 × 3 are denoted as $\mathrm{a}$–$\mathrm{i}$. Figure 4a visualizes a typical case of sampling points in DConv. The parameters of offsets are generated randomly in network initialization due to no explicit constraints applied on the corresponding offsets, leading to inadequate feature aggregation.

The offsets of the deformable convolution are decoupled into the product of the initial offset and the scale to constrain the learning of the initial offsets. As shown in Fig. 4b, an additional loss is proposed to ensure that the value of the predicted scale map is close to 1, while the value of the pre_offset map should be as close to 0 as possible. After that, the value of the final offset map is constrained to 0. Since the value of the predicted offset map is close to 0, it is easy to train as a traditional convolution. As $\lambda$ decreases during the training process, the values of the scale map and the pre_offset map are affected by the loss of the density map ${\mathcal{L}}_{den}$. Gradually, the most suitable value for the task can be learned from the offsets, and the feature aggregation is more sufficient, as shown in Fig. 4c. Therefore, the potential of deformable convolution is freed to improve the performance of training. In the experiment, the aforementioned process is supported by the following expression:

5 $${\lambda }_{T}={\lambda }_{0}k\left[\frac{\left(T-m\right)}{ \, \text{step }}\right]+1$$

For epoch *T*, the weighting coefficient is ${\lambda }_{T}$, and the initial weight, decay rate and evaluation epoch are denoted by ${\lambda }_{0}$, *k* and *m*, respectively.

### Net architecture

**Figure 5:**
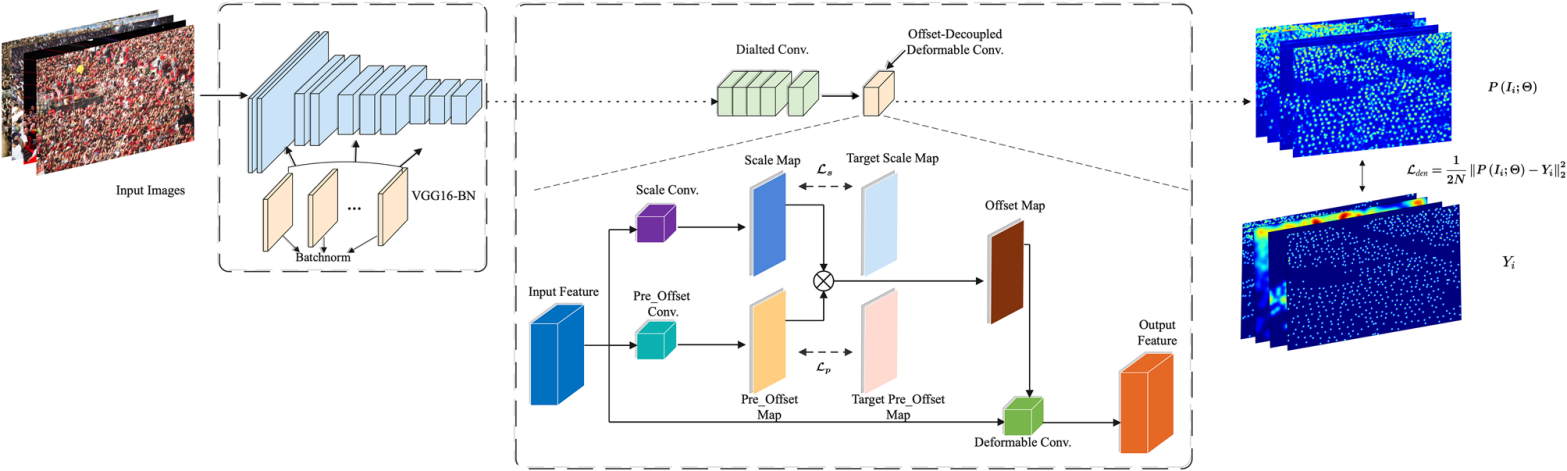
The architecture of the ODConv network. The backbone of CSRNet is replaced with VGG16-BN by inserting the batch normalization layer after each dilated convolution. Then, the last layer of dilated convolution is replaced by offset-decoupled deformable convolution, and the network is defined as our ODConv.

The architecture of the network is represented in Fig. 5.

CSRNet^18^ is used as the backbone network. In particular, the batch normalization layer is integrated after each convolution layer to boost the robustness of training with cropped images. To validate the effectiveness of ODConv, the last layer of dilated convolution is replaced with DConv and ODConv, denoted as CSRNet⊕ and CSRNet (ODConv), respectively. In the following section, the effectiveness of our ODConv is validated.

### Ethics statement

**Figure 6:**
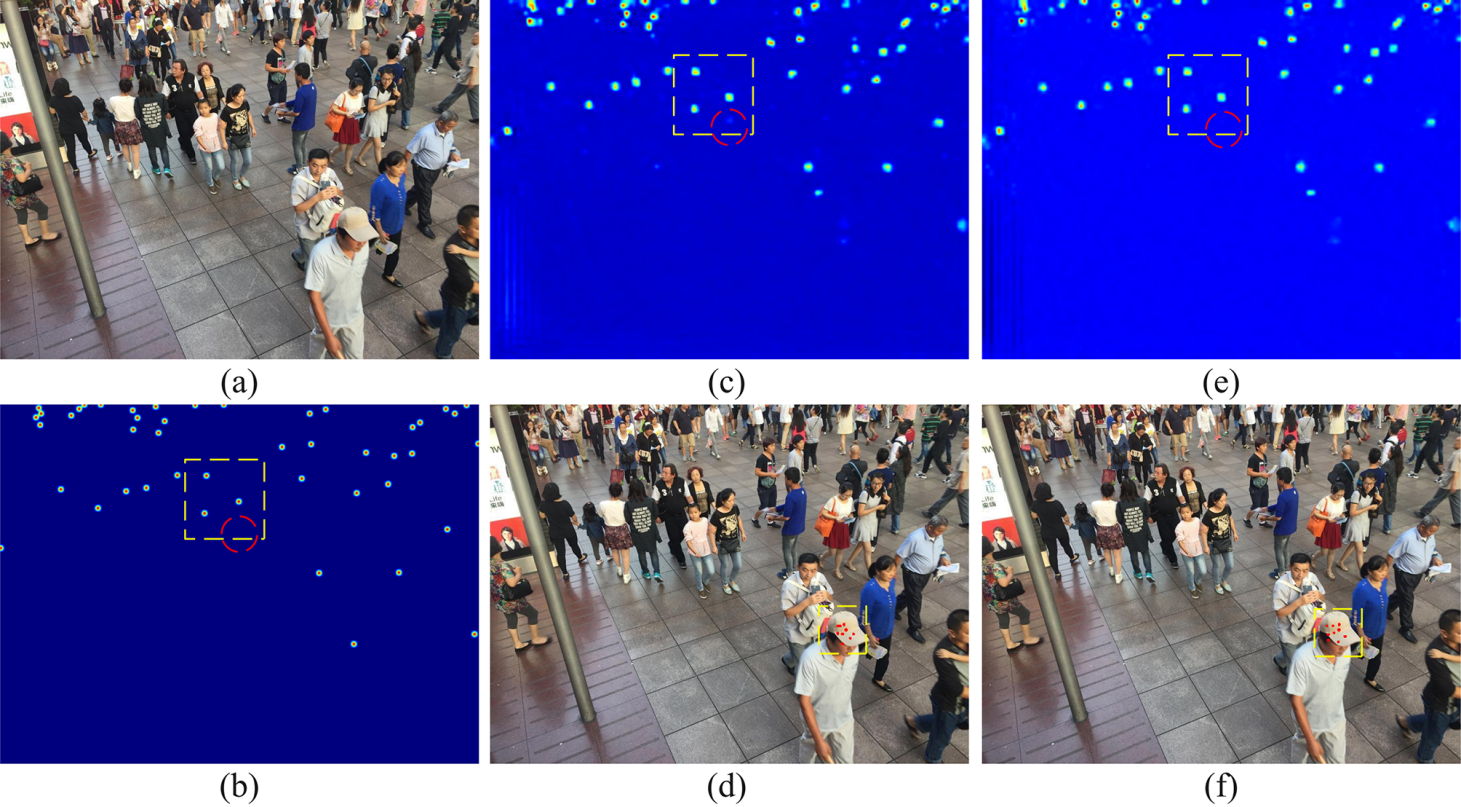
Visualization of an image from the ShanghaiTech B dataset. The first column shows one of the samples and its ground truth devoted as (**a**) and (**b**), respectively. The predicted density map in DConv and ODConv is shown in (**c**) and (**e**), and the visualization of offsets of DConv and ODConv is presented in (**d**) and (**f**).

The authors declare that the images that could lead to human identification (in Figs. 1a and 6a,d,f) are all from an open-source dataset called ShanghaiTech Part B. Many state-of-the-art crowd counting methods are tested on this dataset. All subjects and/or their legal guardian(s) provide informed consent for the publication of identifying information and/or images in an open-access online publication.

## Implementation details

In this section, the evaluation metric and datasets are introduced, and then the implementation details are described.

### Evaluation metrics

The same methods as in the prior work^29,30^ are used in the evaluation metrics, which adopt mean absolute error (i.e., MAE) and mean square error (i.e., MSE) to verify accuracy. MAE and MSE are shown in Eqs. (6) and (7), respectively:

6 $$MAE=\frac{1}{N}{\sum }_{i=1}^{N}\left|{Y}_{i}-\widehat{{Y}_{i}}\right|$$

7 $$MSE=\sqrt{\frac{1}{N}{\sum }_{i=1}^{N}{\left|{Y}_{i}-\widehat{{Y}_{i}}\right|}^{2}}$$

where *N* is the number of samples and ${Y}_{i}$ and $\widehat{{Y}_{i}}$ are the ground truth and predicted counts, respectively.

### Datasets

On four popular datasets, ShanghaiTech Part A, ShanghaiTech Part B, UCF-QNRF, and UCF_CC_50, the ODConv and other state-of-the-art approaches are evaluated. Furthermore, ablation experiments are carried out on UCF-QNRF and UCF_CC_50.

**UCF_CC_50 **^15^. The UCF_CC_50 dataset contains 50 images with varied crowd densities. The range of counts in each image varies from 94 to 4543, with an average number of 1280. To increase the amount of data and test accuracy, we divided the dataset into 5 groups according to fivefold cross-validation.

**UCF-QNRF **^31^. UCF-QNRF is characterized by high resolution and high crowd density. It contains 1535 images, with an average of 814 head annotations per image, and there are 12,865 people in the most crowded image. Compared with ShanghaiTech B, UCF-QNRF has more images for testing, 334 in total, and the rest are training images.

**ShanghaiTech A and B **^23^. Most of the pictures in this dataset, which is 185 divided into Part A and Part B, come from the internet and the streets of Shanghai. Part A contains 482 images, and Part B contains 716 images. The difference between the two parts is that the resolution of Part B is higher and the number of images is larger.

### Training details

First, the backbone of CSRNet^18^ is replaced with VGG16-BN. In particular, since the batch normalization layer is popularly utilized in crowd counting, similar to most crowd counting approaches^32–36^, it is integrated after each convolution layer in our backbone to boost the robustness of training with cropped images. After replacement, the overall network is regarded as the baseline of the experiment, which is called CSRNet⊕ . Finally, the last layer of dilated convolution in the baseline is replaced by offset-decoupled deformable convolution, and the network is defined as our ODConv.

In the experiments, a fixed 7 × 7 Gaussian kernel is utilized to generate density maps. The Adam optimizer^37^ is adopted with a fixed learning rate of 1*e *− 3. All images are resized to 400 × 400. The parameters ${\lambda }_{0}$, $k$, $m$*,* and *step* are set to 4, 0.2, 50, and 50, respectively. With different parameter combinations, 400 epochs are trained in 2 days for each experiment.

## Experimental results

In this section, the proposed ODconv is compared with state-of-the-art methods on the four abovementioned datasets. In addition, an ablation study is conducted to demonstrate the superiority of the parameters utilized in the experiments.

### Evaluations and comparisons

**UCF-QNRF and UCF_CC_50**. Significant gains of 91.9 and 159.3 MAE, respectively, are obtained in the test results with our proposed method on the datasets UCF-QRNF and UCF_CC_50, as shown in Table 1. In particular, our method achieves 3.6 and 15.3 MAE gain over CSRNet⊕ on the premise that the performance of the baseline is greatly improved, which shows the effectiveness of decoupling learning offsets in dense scenes.

**Table 1:** Comparisons on UCF-QNRF and UCF_CC_50.

Methods	Venue&Year	UCF-QNRF		UCF_CC_50	
		MAE	MSE	MAE	MSE
MCNN^23^	CVPR2016	277	426	377.6	509.1
CMTL^38^	AVSS2017	252	514	322.8	341.4
Switch-CNN^39^	CVPR2017	228	445	318.1	439.2
CSRNet^18^	CVPR2018	–	–	266.1	397.5
SANet^40^	ECCV2018	–	–	258.4	334.9
PSDDN^41^	CVPR2019	–	–	359.4	514.8
DensityCNN^19^	TMM2020	101.5	186.9	244.6	341.8
DENet^42^	TMM2020	–	–	241.9	345.4
CSRNet⊕ (Baseline)	–	95.5	165.3	174.6	237.0
Ours (ODConv)	–	91.9	163.1	159.3	233.6

**ShanghaiTech A and B**. As shown in Table 2, compared with the state-of-the-art crowd counting algorithm, our method performs well, which shows 215 the advantage of offset-decoupled deformable convolution in experiments. For example, on the ShanghaiTech Part A dataset, our method achieves the best 62.3 MAE, and the performance is improved by 1.8 MAE compared with the baseline CSRNet⊕ . Our method also achieves the best result of 8.3 MAE on ShanghaiTech Part B, with a 0.7 MAE decrease compared to the baseline, indicating that our method meets the expectations.

**Table 2:** Comparisons on ShanghaiTech A and B.

Methods	Venue&Year	ShanghaiTechA		ShanghaiTechB	
		MAE	MSE	MAE	MSE
MCNN^23^	CVPR2016	110.2	173.2	26.4	41.3
CMTL^38^	AVSS2017	101.3	152.4	20.0	31.1
Switch-CNN^39^	CVPR2017	90.4	135.0	21.6	33.4
CSRNet^18^	CVPR2018	68.2	115.0	10.6	16.0
SANet^40^	ECCV2018	67.0	104.5	8.4	13.6
PSDDN^41^	CVPR2019	65.9	112.3	9.1	14.2
DensityCNN^19^	TMM2020	63.1	106.3	9.1	16.3
DENet^42^	TMM2020	65.5	101.2	9.6	15.4
CSRNet⊕ (Baseline)	–	64.1	104.6	9.0	15.3
Ours (ODConv)	–	62.3	103.4	8.3	13.7

### Ablation study

**Figure 7:**
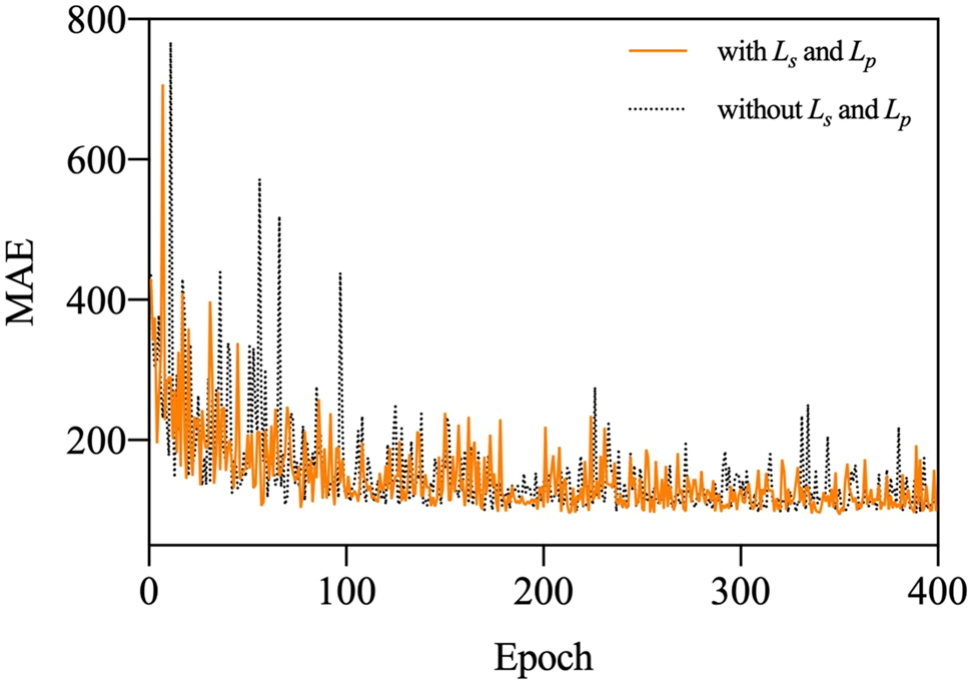
Training curves of ODConv and DConv on the UCF-QNRF. The training process with ${\mathcal{L}}_{\mathrm{s}}$ and ${\mathcal{L}}_{\mathrm{p}}$ is indicated by the orange solid line, and another gray dotted line presents the training process without ${\mathcal{L}}_{s}$ *and *${\mathcal{L}}_{p}$*.*

In this section, the influence of the number of deformable layers in our network is first demonstrated. In addition, the validity of offset-decoupled deformable convolution is confirmed by the visualization of offsets in Fig. 6. To indicate the effectiveness of the proposed method, the training curve is shown in Fig. 7. Finally, the most suitable value of the weight and decay rate in the constrained loss of scale map is validated by comparison.

#### Influence of the number of deformable layers

Dilated convolution, which is integrated into the architecture of CSRNet, is gradually changed into deformable convolution. Then, each layer of deformable convolution is replaced with ODConv. As the performance shows in Table 3, the performance is better when the number of deformable layers is smaller. The peak values of the baseline are from 172.9 to 187.4 MAE, while the trend in performance of ODConv is similar, which is from 159.3 to 179.3 MAE on UCF_CC_50. Thus, it is more effective to merely replace the last layer of dilated convolution with DConv and ODConv in the network.

**Table 3:** Effects of the number of deformable layers on UCF_CC_50.

Deformable layers	CSRNet⊕		Ours (ODConv)	
	MAE	MSE	MAE	MSE
1	174.6	237.0	159.3	233.6
2	172.9	268.3	162.5	232.1
3	175.4	233.3	176.4	264.7
4	182.7	271.2	179.3	269.4
5	187.4	266.5	169.8	261.4
6	174.9	256.2	168.5	232.2

#### Visualization of decoupled offsets

The sampling points of DConv and our proposed ODConv are shown in Fig. 6. The results of experiments show that our ODConv exceeds deformable convolution, while the effectiveness of our method is also verified by the visualization of offsets. Compared with the baseline, the sampling points of ODConv are not disordered or stacked but evenly distributed over the heads, validating that the feature aggregation in ODConv is more sufficient than the baseline.

#### The training curves of ODConv and DConv

To illustrate the effectiveness of ODConv compared with DConv, we adopt a metric called the mean absolute error (i.e., MAE). The curves of the comparison with or without ${\mathcal{L}}_{s}$ and ${\mathcal{L}}_{p}$ in training are shown in Fig. 7. In the initial 100 epochs, the sampling points of the deformable convolution without ${\mathcal{L}}_{s}$ and ${\mathcal{L}}_{p}$ are disorderly stacked, weakening the effectiveness of feature extraction. By comparison, the offsets can gradually learn the value that is most suitable for the task, so the improved performance is achieved in our proposed ODConv.

#### The weight and decay rate in the constrained loss of scale map

To reduce the impact caused by the variance of the sampling points, additional loss is added to the predicted scale map to make it as close as possible to 1.

**Figure 8:**
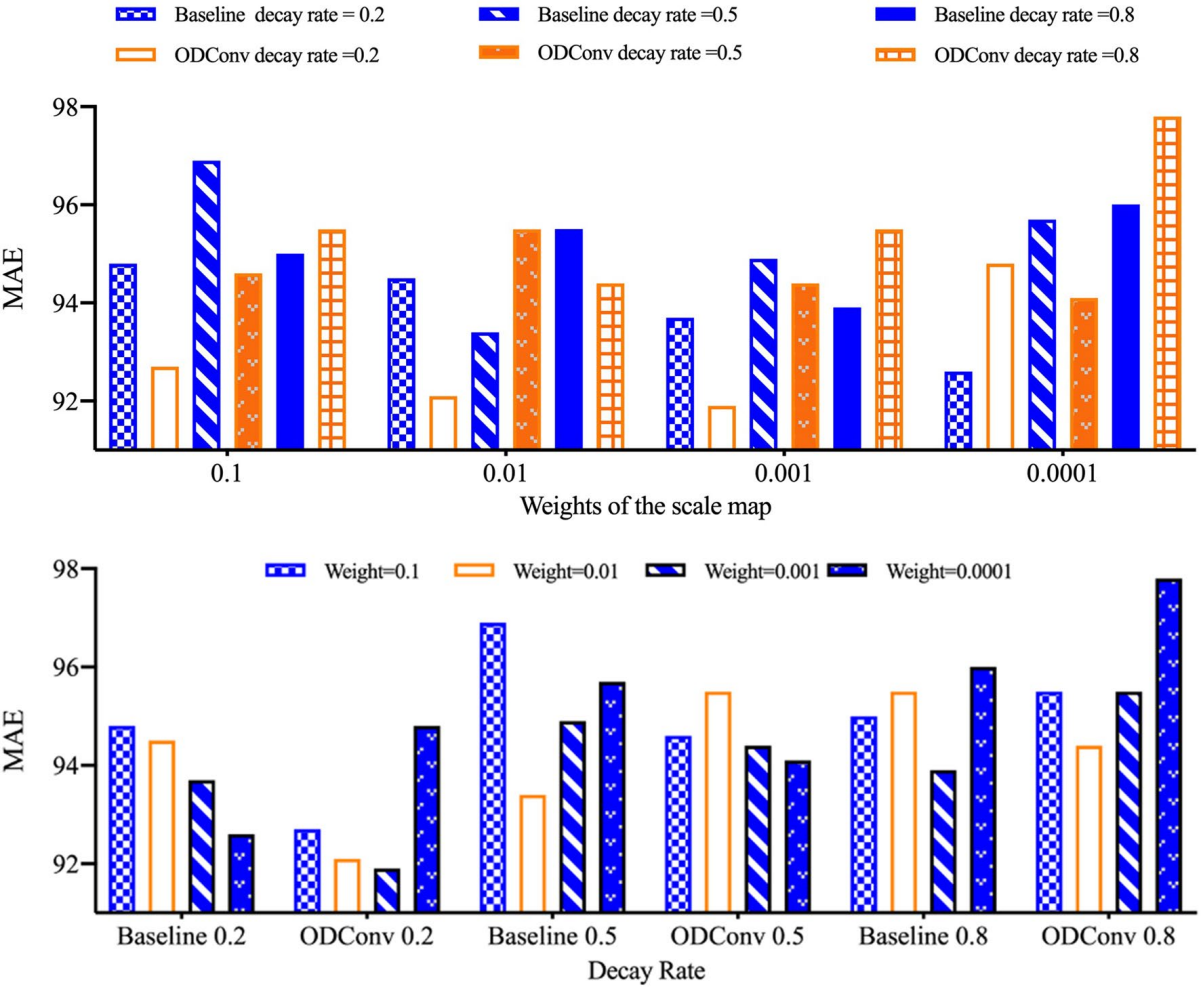
The comparisons of DConv and our ODConv with different weights of the scale map and decay rates.

With the progress of training, ${\lambda }_{0}$ gradually decreases. The loss of the density map ${\mathcal{L}}_{den}$ acts on the values of the scale map and the pre_offset map. By conducting the ablation study, it is verified that when the weight of the scale map is 0.001 and the attenuation rate is 0.2, offsets can gradually learn the most suitable value for the task. Therefore, the potential of deformable convolution is further exploited. The detailed trend of the data is shown in Fig. 8.

#### T-test analysis

**Figure 9:**
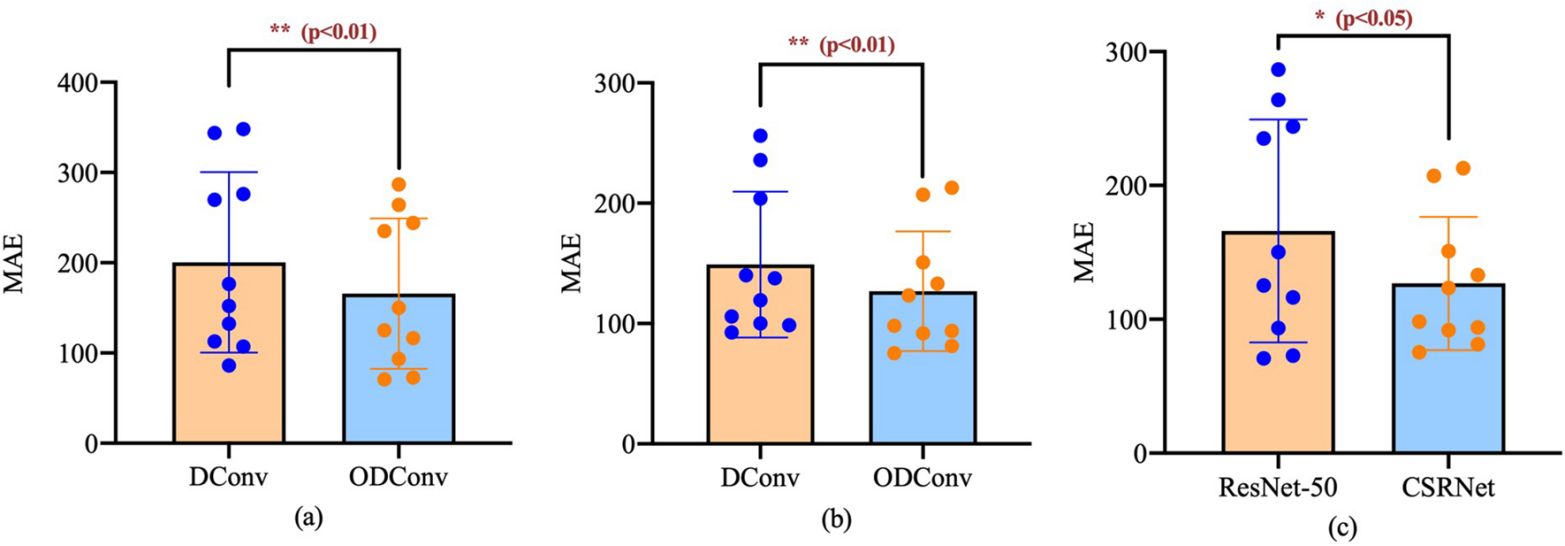
Comparisons of the ODConv and DConv on the ResNet-50 and CSRNet are shown in (**a**) and (**b**), respectively, and comparisons of the ODConv on the CSRNet and ResNet-50 are shown in (**c**). The results of the significance level are indicated by the crimson characters on the top of each figure.

To illustrate the robustness of ODConv on other backbones and the effectiveness compared with DConv, the T-test^43^ is adopted in which a p-value is calculated to determine whether the model’s performance is statistically significant. If the p-value is less than 0.05, we could consider that a significant difference exists between the two sets of data analysis. In this section, a new baseline consisting of ResNet-50^44^ is built to verify the extensibility of ODConv. The last layer of dilated convolution of ResNet-50 (backbone) is replaced with Deformable Convolution and Offset-decoupled Deformable Convolution called ResNet-50 (DConv) and ResNet-50 (ODConv), respectively. Then, the UCF_CC_50 dataset is divided into ten subsets, and tenfold cross-validation is performed. A graphical representation of the results is shown in Fig. 9. In Fig. 9a, the MAE-values of the ResNet-50 (DConv) and ResNet-50 (ODConv) are compared and analyzed using the paired T-test to determine whether ODConv can make a significant difference in results. The utilized ODConv can make a significant difference compared with the DConv, as indeed suggested by the p-value (***t*** = 3.513, ***p*** = 0.0066 < 0.05). Then, the p-value (***t*** = 4.167, ***p*** = 0.0024 < 0.05) on CSRNet (DConv) and Ours (ODConv) in Fig. 9b can also verify the above statement. Significant differences can also be seen in ResNet-50 (ODConv) and Ours (ODConv) in Fig. 9c, with a p-value of 0.0194 (***t*** = 2.840, ***p*** = 0.0194 < 0.05), indicating that ODConv can make significant differences on different backbones.

## Conclusion

In this paper, an offset-decoupled deformable convolution (ODConv) is proposed. Compared with the original method, the superiority of ODConv shown on constrained offsets. The offsets of sampling points in ODConv are decomposed, and the constraints are added to the offsets, decreasing the confusion or stacking of sampling points. As an example, the crowd counting results show that our ODConv can effectively improve the performance with little extra computational burden.

## Data Availability

All data used in this paper can be requested from the corresponding author.
